# Alcohol Consumption Under the Shadow of Coronavirus Pandemic

**DOI:** 10.31661/gmj.v9i0.1922

**Published:** 2020-09-09

**Authors:** Amir Adibi, Aliashraf Mozafari, Hadis Jamshidbeigi, Tayebe Jamshidbeigi, Ali Sahebi

**Affiliations:** ^1^Department of Child and Adolescent Psychiatry, Ilam University of Medical Sciences, Ilam, Iran; ^2^Clinical Research Development Unit, Shahid Mostafa Khomeini Hospital, Ilam University of Medical Sciences, Ilam, Iran; ^3^Department of Internal Medicine, Ilam University of Medical Sciences, Ilam, Iran

**Keywords:** Alcohol, Alcohol Consumption, COVID-19, Coronavirus, Pandemic

## Dear Editor,

On 31 December 2019, several cases of COVID-19 were reported by national authorities in China. There were soon detected in several other countries [1]. Given that COVID-19 is a new disease with worldwide devastating effects, it is understandable that its emergence and spread cause considerable confusion, anxiety, and fear among the people. Despite the supportive evidence for realities change, there still exists many rumors and misinformation to ensure the prevention and management of COVID-19 in society [2]. According to the World Health Organization (WHO], the disease is transmitted through touch respiratory droplets and close contact methods. Respiratory droplets produced during normal conversation, cough, or sneeze may be important in transmitting disease, especially indoors. Infection can also occur if droplets land on surfaces like a doorknob, where persons can touch lingering virus particles and transfer them to their mucous membranes. Evidence suggests that people infected with COVID-19 have the virus in their stool[3]. To prevent the spread of this disease, such as frequent handwashing with soap and water is critical. Besides, using alcohol-based hand rub is a good way to clean and prevent the spread of COVID - 19. There are wide ranges of disinfectant available, but alcoholic disinfectants are the best disinfectants for viruses [4]. Alcohol consumption contributes to a range of health, social and behavioral problems, including liver cirrhosis, mental illness, various types of cancer, pancreatitis, and fetal damage in pregnant women. A recent review conducted from around the world shows that alcohol consumption can lead to at least 61 different types of health problems such as injuries, violence, cancer, liver diseases, and deaths [5]. In Iran, misinformation and rumors have spread that alcohol consumption does disinfect the mouth or provides protection for COVID-19, as a result, many patients referred to the emergency departments due to alcohol poisoning as a failed attempt to prevent COVID-19. Since there is illegal for Iran’s citizens to produce, buy, or sell alcoholic beverages, many of these people have fallen victim to the consumption of illicitly produced alcoholic beverages [6]. As of March 28, 2020, in Iran, a total of 1000 people had poisoned and 300 people had died by methanol alcohol intoxication [7]. The Covid-19 pandemic sparked widespread rumors and misinformation swirled through cyberspace. The spread of rumors might cause panic and concern among the people. Unfortunately, some people, wrongly believed that drinking alcohol causes their immunity to the disease, was poisoned and hospitalized and even died. Spreading rumors and misconceptions in times of crisis can lead to misbehavior among people that profoundly affect their physical and mental health status. If this is not taken into account by the authorities and the people, it will cause irreparable damage to the individual and society. To prevent these injuries, when communities are facing a crisis, timely and accurate news and information must be provided to the people, and the consequences of the false information must be taken into account.

## Conflict of Interest

 None declared.
